# Electrospun nanofibers for the fabrication of engineered vascular grafts

**DOI:** 10.1186/s13036-019-0199-7

**Published:** 2019-11-11

**Authors:** Sonia Fathi Karkan, Soodabeh Davaran, Reza Rahbarghazi, Roya Salehi, Abolfazl Akbarzadeh

**Affiliations:** 1 Department of Medical Nanotechnology, Faculty of Advanced Medical Science, Tabriz University of Medical Sciences, Golgasht St, Tabriz, Iran; 2 Drug Applied Research Center, Tabriz University of Medical Sciences, Tabriz, Iran; 3 Student Research Committee, Tabriz University of Medical Sciences, Tabriz, Iran; 4 Stem Cell Research Center, Tabriz University of Medical Sciences, Tabriz, Iran; 5 Department of Applied Cell Sciences, Faculty of Advanced Medical Sciences, Tabriz University of Medical Sciences, Golgasht St., Tabriz, Iran; 6 Tuberculosis and Lung Disease Research Center, Tabriz University of Medical Sciences, Tabriz, Iran

**Keywords:** Electrospun nanofibers, Engineered vascular grafts, Angiogenesis, Regenerative medicine

## Abstract

Attention has recently increased in the application of electrospun fibers because of their putative capability to create nanoscale platforms toward tissue engineering. To some extent, electrospun fibers are applicable to the extracellular matrix by providing a three-dimensional microenvironment in which cells could easily acquire definite functional shape and maintain the cell-to-cell connection. It is noteworthy to declare that placement in different electrospun substrates with appropriate physicochemical properties enables cells to promote their bioactivities, dynamics growth and differentiation, leading to suitable restorative effects. This review paper aims to highlight the application of biomaterials in engineered vascular grafts by using electrospun nanofibers to promote angiogenesis and neovascularization

## Introduction

**Fig. 1:**
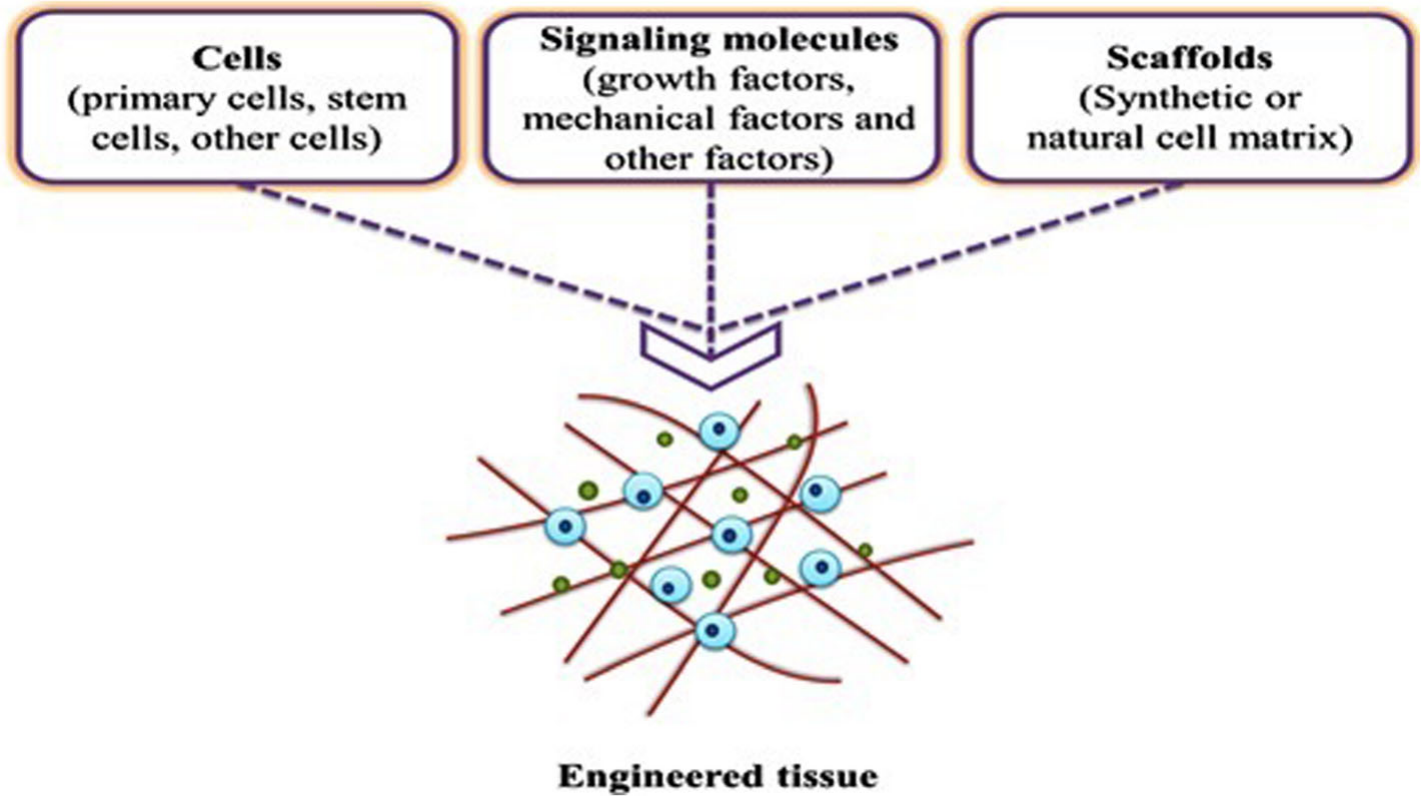
The schematic of three key components in tissue engineering, involving scaffolds, cells and growth factors

Cardiovascular disease is one of the most important issues contributes to human death globally [1]. In cardiovascular medicine, surgical approach and transplantation are often desired therapeutic options to care for patients with pathological conditions [2]. Peripheral vascular disease is touted as a common circulatory disorder that reduces blood nourishment and ultimately leads to the ischemic condition [3]. In the circumstances with the complete vessels obstruction, vascular transplantation and bypass surgery are highly recommended [4]. At the moment, vascular grafts are currently prepared by vascular sections of the patient’s body or from an appropriate donor [5]. Calling attention, vascular transplantation is not often sufficient to meet patient needs due to limited vascular resources. Also, the risk of thrombosis, infection, and rejection of the vascular transplant are very likely in these conditions [6–8]. In line with these conditions, the need for transplantation of artificial vessel is felt more than ever. In this regard, novel tissue engineering technology can be used for vascular reconstruction. So far, the vascular tissue engineering for large vessels had promising successes for vessels structure with a diameter of 6 mm, but the reconstruction of structures below this value is faced with several challenges. The provision of tubes with suitable resistance to the pressure and cyclic loading of blood that couple with the host vessels possessing antithrombotic activity are at the center of attention [9, 10]. Tissue engineering is interdisciplinary scientific modalities uses different methodologies to circumvent problems associated with tissue injury or organ loss. In terms of structural components, tissue engineering consists of three main components as follows; scaffolds act as an underlying substitute with an applicable function to the ECM, growth factors that promote intracellular signaling effectors and distinct cell types as the main component of the tissue structure (Fig. 1) [11]. To achieve this end, tissue engineering usually starts with the fabrication of a specific matrix composed of multiple suitable components from a natural source (proteins) and synthetic materials (polymers). The constructs should have potential to imitate almost all aspects of the natural microenvironment. However, scientific challenges still exist in the fabricated substrates parameters and values associated with the creation of environment comparable to in vivo condition. Up to the present, the application of electrospun-nanofibers, as a scaffold, is being popular in tissue engineering. To fabricate electrospun-nanofibers, multiple nano-sized scaffolding can be designed in 2D, and 3D with a porous structure to attain a high surface area-to-volume ratio, allowing cells to maintain juxtacrine interaction with each other [12]. All of these features have contributed to the emergence of comprehensive high throughput restorative effects in which electrospun nanofibers have been suggested as a novel modality with satisfactory outcomes in the restoration of cutaneous tissue, blood vessels, cartilage, bone, etc. [13, 14].

The current review article tried to collect novel data in the fabrication of vascular grafts, in particular, the electrospinning approach, for the induction of angiogenesis and blood nourishment. We also scrutinized cellular response and clinical promise of engineered vascular grafts produced by electrospun nanofibers.

## General blood vessels structure

In general, blood circulates through the body via closed different vessels such as arteries, veins, and capillaries. Arteries have the potential to transport blood from the cardiac tissue to other organs, plus the systemic arteries are transporting oxygen-rich blood to the tissues. Accordingly, the arteries need an elastic wall to counteract the pressure effected when the ventricular and muscle contraction to enable better contraction to aid move the blood [15, 16].

**Fig. 2:**
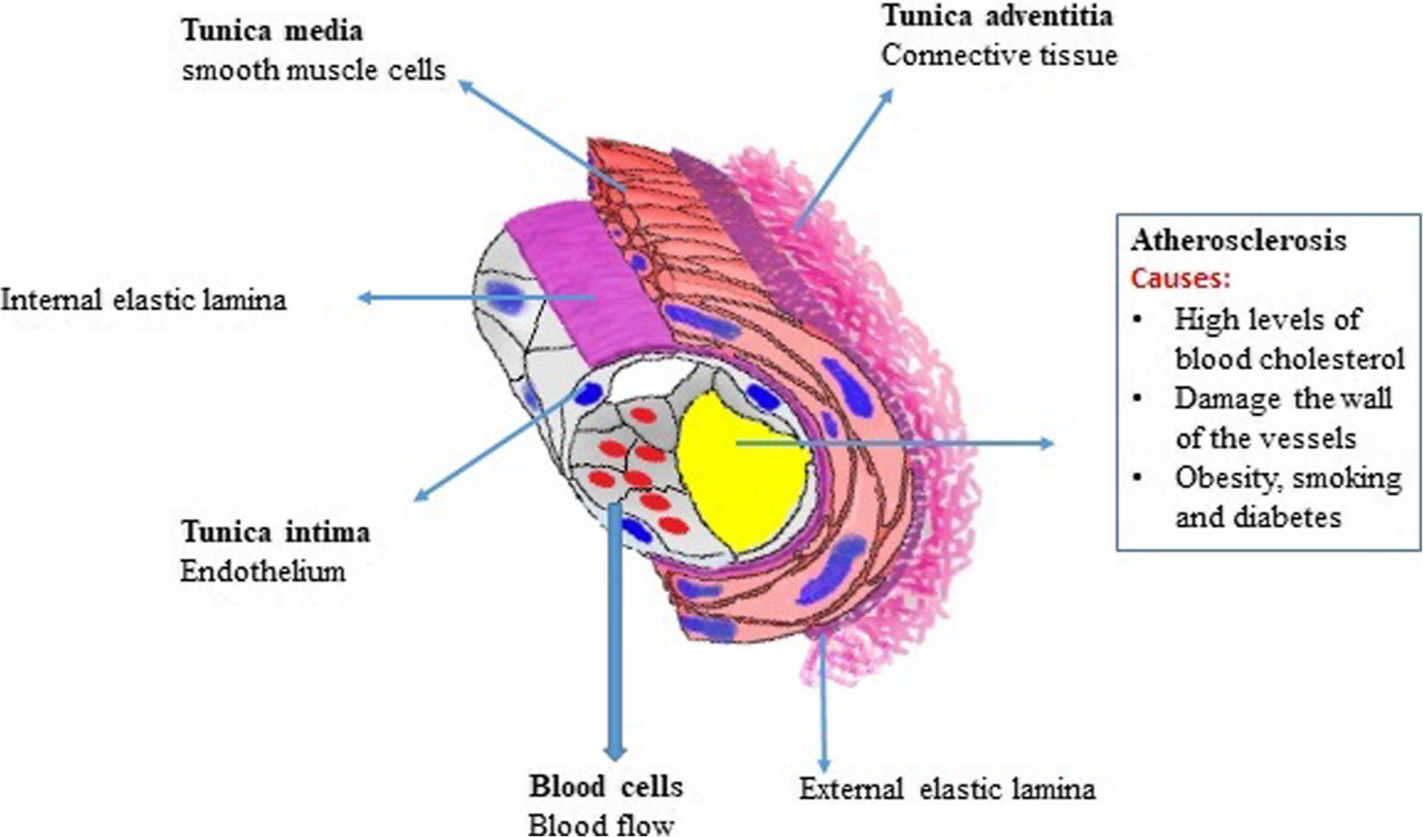
The schematic of principal structural features of the larger blood vessels as seen in a muscular artery

The veins are responsible for returning blood to the heart. Most often, the vein returns the deoxygenated blood from various tissues to the heart, with the exception of the pulmonary vein and the umbilical vein that returns oxygen-carrying blood to the heart. The vein is less muscular than the artery and has valves that prevent blood backflow [17]. The smallest and most abundant vessels in the body are capillaries that form the connections between the arteries and veins. Since the capillaries have a thin wall and the blood flow in them is slow, so the swapping of materials between blood and cells is done in the capillaries [18]. From a histological point of view, vessels contain three distinct zones from luminal to outer surface including tunica intima, tunica media and tunica adventitia (Fig. 2) [19]. A term lumen refers to the interior space of the vessel which is surrounded by the vessel wall. The layer tunica intima is arteries inner layer that paved with a single-layer column of ECs lining subendothelial layer and is in direct contact with blood flow. For the consistency and resistance of tunica intima against mechanical stress, the endothelial layer is supported by the basal internal elastic lamina. Internal elastic lamina acts as a border and isolates the tunica intima from the lower middle layer named tunica media. To be informed, tunica media is the middle layer of vessels wall consisted of a regular circle of rows of α-SMCs, fibroblasts surround by elastic fibers in collagenous bed. The outer layer, tunica adventitia, is composed of fibroblasts, collagen and distinguishable from tunica media by an external elastic lamina [20, 21]. In large size vessels with a thick wall, the penetration of blood substances is impossible while capillaries composed of a single-cell thin layer have the potential to exchange materials reciprocally between blood and neighboring tissues [9, 22, 23]. Based on the scientific reports, two forces interact with the exchange of materials through the capillary walls. As previously mentioned in the literature, blood pressure is high inside capillaries and thus provides pressure for the penetration of materials into the interstitial fluid. In contrast, the existence of osmotic force originated from by plasma proteins forces the transportation of liquid phase and metabolic byproducts from tissues (interstitial fluid) to the capillaries. Of note, the amount of this pressure is high in vessels context compared to the interstitial fluid [15, 24, 25]. The biomechanical and biophysical properties of the vessels are related to the entity of ECM enclosed vascular cells. Along with this issue, experiments have shown that cell differentiation, migration, and polarization are under the influence of ECM and the quality and quantity of cell surface glycoconjugates [26, 27]. Therefore, the existence of a unique ECM structure with prominent mechanical properties makes blood vessels suitable for circuit liquid phage. Such mechanical features such as elasticity, compressibility, tensile stiffness, and viscosity are provided by elastin, proteoglycans, and collagens [28].

## Tissue engineering of blood vessels

### Tissue engineering

**Fig. 3:**
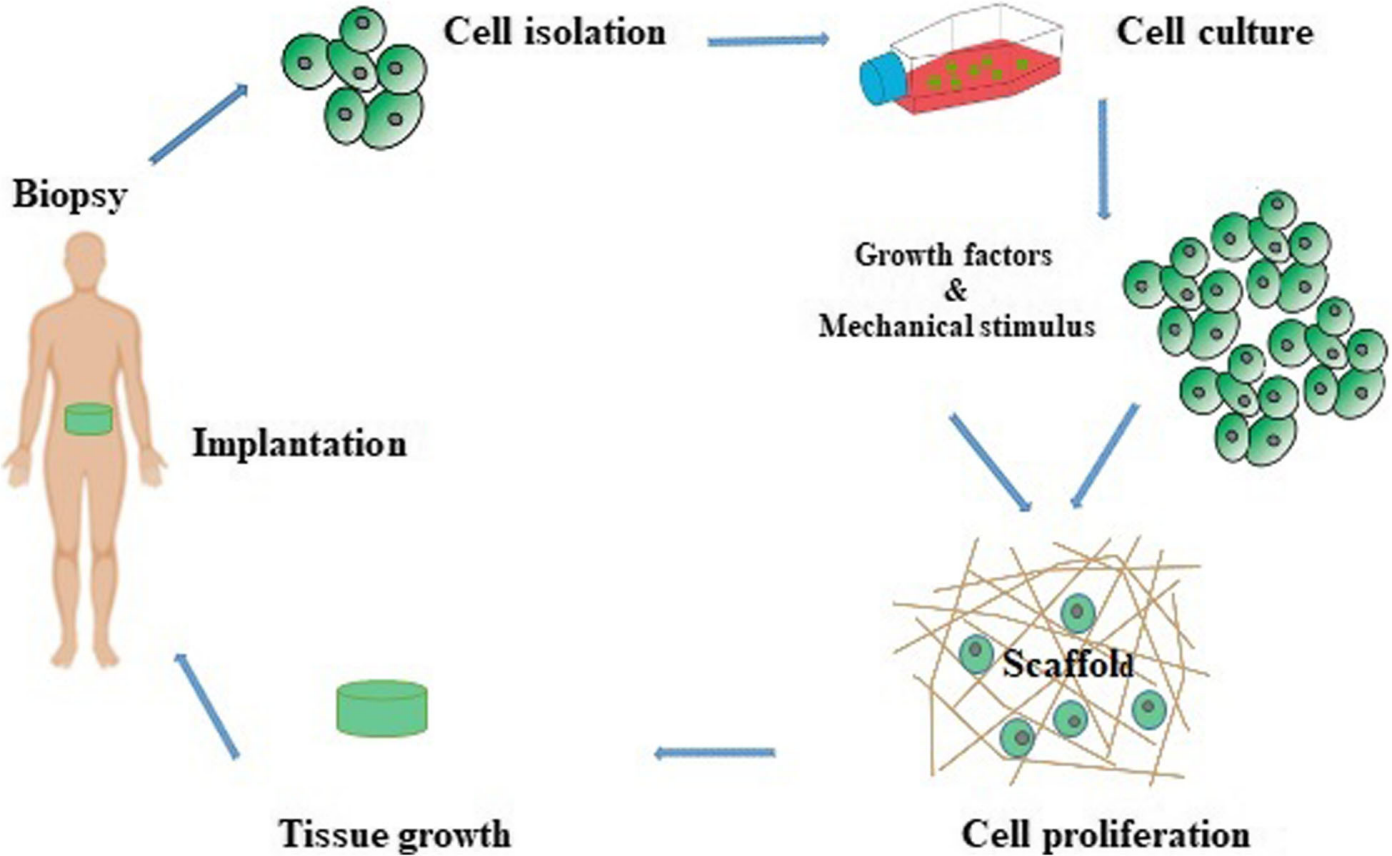
A schematic illustration of the stages of tissue engineering and tissue implantation

In spite of human body vulnerability to injury, it has a unique strength and capacity for self-repair. Along with the development and progress of biomaterial engineering and regenerative modalities, the dream of tissue replacement seems achievable in the near future [29]. In 1900, Carell introduced the term “tissue engineering” for the first time. By virtue of the introduction of a de novo method for vascular anastomosis, the implication of modern tissue transplantation was raised [30, 31]. In short, tissue engineering means the in vitro development and modulation of distinct molecules and cells in natural and synthetic constructs with the purpose of replacing and repairing the damaged tissue. In this modality, porous materials are fabricated as suitable ECM and underlying substrates for the promotion of cell dynamic growth. To increase cellular adaption, various growth factors and cytokines could be conjugated to the substrates. In a better word, the concept of tissue engineering encompasses the spatial support of cells with a prominent physical feature in a 3D milieu. Commonly, the scaffolds mainly consist of synthetic or natural materials (collagen, elastin, and fibrin) acts as basal scaffolds, anchorages for cells or that cells can orient and acquire functional maturation for cultivation in a suitable niche [29, 32]. After approval of proper cell growth in the porous scaffolds, the constructs could be transplanted to the target sites in vivo. Angiogenesis develops into scaffolds to nourish the transplanted cells and to prohibit cell death [29, 33] (Fig. 3). Commensurate with these statements, the promotion of vascularization into the transplanted construct seems to be vital for successful tissue engineering and repair. Once the engineered scaffolds are transplanted into in vivo condition, direct contact of scaffolds with the microenvironment allows the recruitment of diverse ions, proteins, polysaccharides, enzymes and different types of cells as well. Early researches in the mid-twentieth century were focused on the development of bioconversion materials to result in minimal host response, inactive blood transport, and minimal interaction with neighboring tissues. To this end, numerous synthetic materials are extensively available, for example, Silicone and Teflon which not exactly applicable for medical proposes. Today, many biomaterials are designed to maintain the reciprocal interaction between proteins and cells at the molecular levels in a very precise and manageable manner. The main purpose of the development and design of these biomaterials is that the scaffolds should contain chemical or structural information that can imitate cell-cell interaction and control the formation of tissue. Agents such as growth factors and the adhesion Arginyl-glycyl-aspartic acid (RGD) peptide sequences and other molecules with a structure resembling ECM components are highly requested [34, 35].

### Common biomaterials in tissue engineering

The term “biomaterial” means the technology of application materials as scaffolds with an ability to control cellular bioactivities. In addition to the comparable structure of biomaterials to the in vivo condition, these materials must be compatible with the host sites while at the same time stimulating cell signaling by providing extracellular signals. Williams described the term biomaterial as any synthetic or natural substances that include all or part of a living structure and function with the capability to replace with the host tissue (Table 1) [36, 37]. Biomaterial properties must be defined in accordance with the type of cells and organs and transplantation site. The importance of mechanical properties, which is directly related to the target tissue, should not be ignored [38]. Irrespective the specific feature of different scaffolds, all biomaterials should possess biocompatibility and eschew from the immune response [39]. Regarding engineered vessels grafts, thrombogenic properties of these structures should be investigated which are in close contact with blood and coagulation factors. In addition, it should be noted that the biomaterials used in these structures should have an inherent resistance to tolerate and adapt to continuous blood pressure. As a matter of fact, different values such as burst pressure, suture strength and exhaustion must be considered prior to implantation [39, 40].

**Table 1:** List of biomaterials are commonly used for vascular tissue engineering

Biomaterials		
Natural materials	• Collagen	[50, 51]
	• Fibrin	[52, 53]
	• Hyaluronic acid (Hyaff)	[54, 55]
	• Bacterial cellulose (BC)	[56, 57]
	• Silk fibroin (SF)	[58, 59]
	• Small intestinal submucosa (SIS)	[60, 61]
	• Alginate	[55, 62]
	• Chitosan	[55, 63]
Synthetic materials	• Extended Poly (tetrafluoroethylene) (ePTFE)	[64, 65]
	• Poly (ethylene terephthalate) (PET)	[66, 67]
	• Polyhedral oligomeric silsesquioxane poly (carbonate-urea) urethane (POSS-PCU)	[68]
	• Polyglycolic acid (PGA)	[69, 70]
	• PGD-caprolactone-lactic acid (PGA-CL/LA)	[71, 72]
	• PGA-poly-4-hydroxybutyrate (PGA-P4HB)	[73, 74]
	• Polyhydroxyalkanoate-PGA (PGA-PHA)	[75]
	• Polycaprolactone (PCL)	[40]

In 1896, the first report of natural vascular transplantation by Jaboulay and Briau was done, although the vascular anastomoses rate was incomplete and thrombosis induced [41, 42]. To date, many artificial techniques have been developed for the fabrication of vascular autografts, which are routinely used in bypass surgery. However, the access of autografts has some limitations. By the discovery of Vinyon N (nylon) by Voorhees and Dacron® by DeBakey, the development of artificial vascular grafts entered a new phase [43]. Despite the fact that transplantation of larger vascular grafts with a diameter of > 6 mm) contributed to prominent high-flow rate, but the development of thrombi and increase of compliance mismatch have been shown in a small diameter vessel [44, 45]. To overcome these limitations, advanced techniques have been developed to increase construct potency, for example, chemical modifications on surfaces with different coating methods and cell plating approach.

Natural biomaterials have superior effects to synthetic counterparts due to the existence of adhesion motifs; however, synthetic materials have a higher mechanical resistance rate [46, 47]. Natural polymers such as collagen, alginate, fibronectin, chitosan, fibrin, and gelatin often provide the moieties for cell adhesion, proliferation and functional differentiation thus attracted a lot of attention. Due to lack of sufficient mechanical properties of natural polymers, it is not reasonable to use them for vascular scaffolds alone. To solve this issue, the combination of natural and synthetic polymers could be an effective strategy [48, 49]. The mixture must be able to provide all or some of the following conditions for cell growth dynamic and mimic in vivo condition.

## Scaffold design

Each tissue consists of ECM and a certain number of cells. The ECM acts as a 3D scaffold to preserve cell-to-cell integrity inside the body. In general, three types of molecules are present in the ECM of all tissues as follows; (a) structural proteins such as collagen and elastin which provide flexibility and strength; (b) protein-polysaccharide complexes to integrate with the structural proteins (proteoglycans); and (c) adhesive glycoproteins such as fibronectin and laminin that connect each cell to the ECM [76, 77]. It is shown that cells interact with the scaffolds after plating on the surface [78]. In addition to the supportive role of the scaffold in maintaining cell adhesion, ECM serves as an appropriate reservoir of water, nutrients, cytokines and growth factors [79]. Meanwhile, the constructs must have good macroscopic and microscopic properties in which these properties not only affect the cell’s life, signaling pathways, growth, proliferation, or organization but also affect the expression of the gene and maintain the cell’s phenotype [80].

### Critical factors in the design of engineered vascular tissue scaffolds

The design of multilayer structures as vascular scaffolds with respect to the natural layers of the normal vessels and physiological activity of each layer could contribute to the fabrication of similar vascular structure while creating more elasticity, and improving mechanical properties [63]. By using the mixture of natural and synthetic polymers and design changes, it enables us to fabricate desirable structures with density, viscoelastic response, and near-to-natural vessels. Despite advances in the design of the scaffolding, there are still scientific challenges over the design of the ideal scaffolds (Table 2). Here, we point the basic features that are expected from engineered vascular scaffolds.

**Table 2:** General properties for scaffolds and challenges

General Properties Of Scaffolds	Challenges	References
Biocompatibility	• Non-controlling degradation of biodegradable polymers in vivo	[103, 104]
	• The toxicity of products produced by the degradation of biocompatible polymers	
	• Low cell seeding efficiency	
Mechanical Properties proper for tissue	• Scaffolding design with mechanical properties proportional to tissue	[29, 38]
	• Mechanical integrity	
	• Protect cells against tensile and pressing forces	
Biodegradability	• The completion of the tissue healing is dependent on the rate of biodegradation	[105, 106]
	• At least toxicity and inflammation	
	• Transmit the tissue growth conduction signals and differentiation	
	• Cell migration	
	• Pore size and porosity proportional to the tissue	
Porous interconnectivity	• At least toxicity and inflammation	[105, 106]
	• Transmit the tissue growth conduction signals and differentiation	
	• Pore size and porosity proportional to the tissue	
	• The possibility of exchanging gases, nutrients and growth factors and waste materials	
	• Cell migration	
Chemical surface and topography	• Cell-cell interactions and cell adhesion, controlling cell function	[91, 107]

#### Biocompatibility

Biocompatibility is touted as one of the most important criteria for evaluating engineering scaffolds. The biocompatible scaffolds lack harmful immunological or pro-inflammatory response after transplantation. The structure and type of transplants must be such that a minimum immunological reaction and recruitment of immune cells happen. The first step in achieving this goal is to apply non-toxic substances. By possessing degradability, transplants will be gently replaced by natural and functional tissues. The use of transplants without these properties contributes to the promotion of immune-mediated reaction and inflammation. In response to these conditions, cell death will happen which in turn exacerbate pathological outcome. Scaffolds should be able to integrate with the host tissue without any harmful immune responses [81, 82]. According to recent data, it was revealed that immune cells notably macrophages could play a critical role after transplantation of scaffolds. Depending on scaffold biocompatibility, macrophages cells could act as double-edged swords and are the frontline of immune defense. During inflammation, macrophages acquire pro-inflammatory phenotype (M1) and after inflammation removal and promotion of healing these cells turn into M2 type. Since scaffold transplantation, phagocytes mainly neutrophils could adhere to the transplant and release numerous hydrolyzing agents and these conditions contribute to initial inflammation. In the next step, macrophages are recalled to the site of transplantation to complete degradation of scaffolds. The non-degradable scaffolds stimulate macrophages to form foreign bodies that are composed of several cells to strengthen degradation capacity. By using appropriate scaffolds with suitable compatibility and degradation rate, macrophages tend to increase polarization to M2 type which could accelerate healing procedure [83].

#### Porosity

The fabricated scaffolds should be porous with interconnected cavities. The high surface to volume ratio in these constructs could support cell growth and facilitate uniform distribution of cells. The formation and growth of microvessels in the structure become more intense [13, 38, 84]. Therefore, the density of cavities inside constructs and interconnectivity are critical for the proper distribution of nutrients and gases and waste products removal [85, 86]. To achieve proper vessel structure, the porosity and mechanical strength of the scaffolds must be optimized [87].

#### Pore size

The size of the pore is an integral feature to engineered constructs. In the case, scaffolds with a smaller pore size may affect the cell loading and block the way of cell penetration. The lack of cell penetration not only decreases total cell density per unit volume of transplant but also it prohibits the production of ECM and vascular penetration into a construct [88, 89].

#### Surface properties

Scaffold surface properties such as surface topography and chemical properties can be effective in cell attachment and proliferation [90, 91]. The adhesion of cells to synthetic surfaces depends on the chemical properties. This index could increases/decreases the number of cells to scaffolds surface which is an inventible character in the engineering of vascular constructs. The existence of topographic properties is cell alignment similar to specific tissue formation and thus controls differentiation rate [92–94].

#### Induction of tissue formation

The induction of tissue formation is a process in which certain cells come together to repair the injured site and differentiate into specific cell lineage [95]. The induction of tissue formation is a phenomenon that can easily be accomplished by using growth factors. Noteworthy, tissue formation can also be induced without growth factors with a special scaffolding design [96].

#### Biodegradability

Biodegradation is one of the features that should be considered in the scaffolding design [97, 98]. In fact, the temporary filling of tissue defects is one of the main goals by using a distinct scaffold [38, 99]. However, the scaffolds must be decomposed over time with tissue regeneration in order to provide the required conditions for the growth of natural tissue. The decomposition rate of each scaffold must be consistent with the growth rate of the tissue. As a matter of fact, the scaffold should be decomposed when the damage is completely restored. This feature potentiates human medicine to fill the natural tissue defects with biodegradable bio-products while will be removed from the body with tissue healing [48, 100, 101]. It should not be forgotten that scaffolds decomposition will be detrimental and toxic when waste byproducts are at high concentrations [101, 102].

#### Mechanical properties

The scaffolds used in tissue engineering should be mechanically stable and able to perform biological tasks in the site of the implant. Mechanical stability depends on the biological materials, scaffolding design and cell metabolism [11, 13, 103].

#### Functionalized scaffolds

**Fig. 4:**
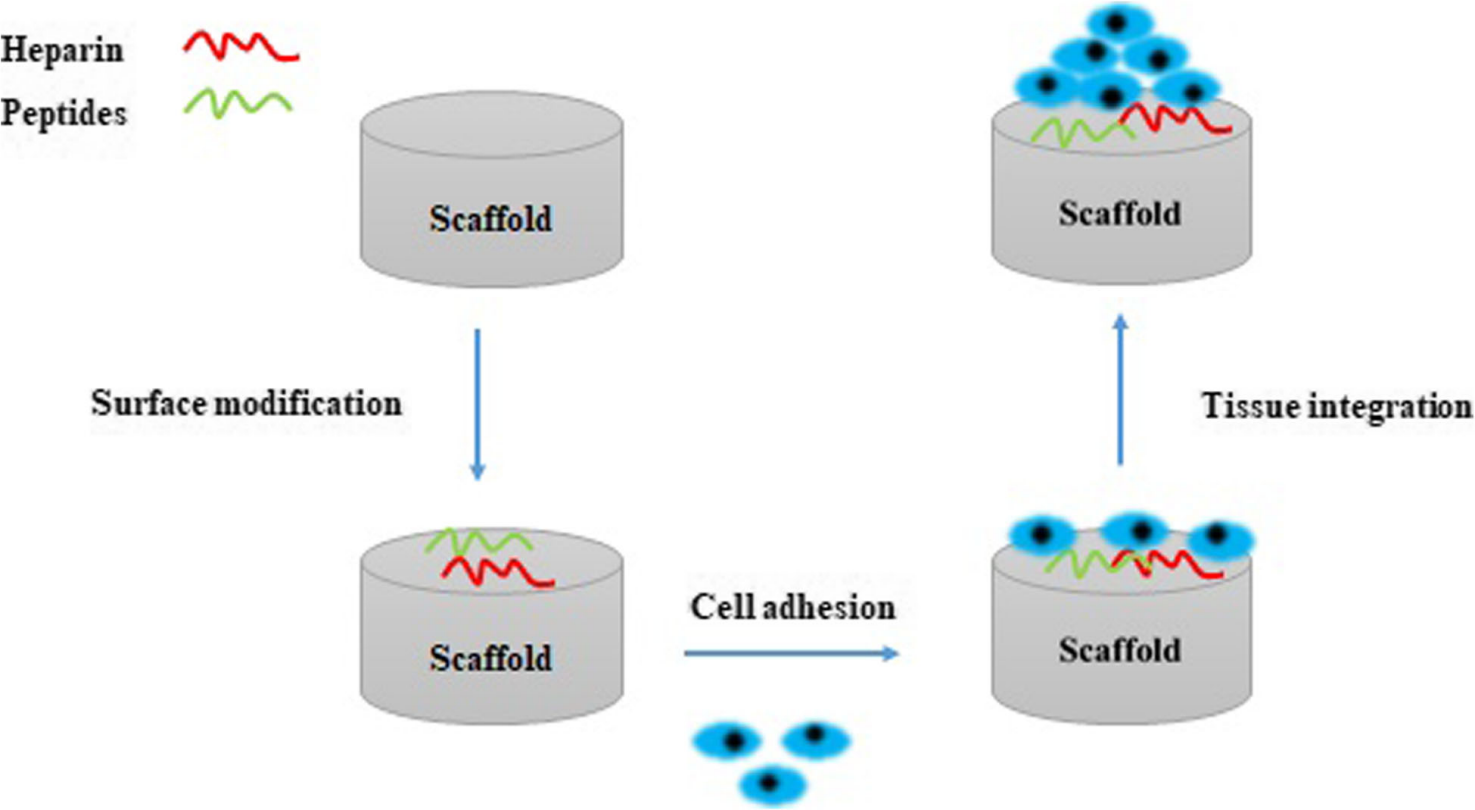
Effect of scaffold functionalization on cell adhesion, tissue regeneration and to prevent blood contractions

This implication stands for a fact that a similar function of constructs is provided, though not a complete function, comparable to the natural counterparts. Modifying the scaffold surface with warfarin, and heparins to prevent blood coagulation is another issue that is considered in the construction of vascular tissue engineering scaffolds. In a better word, engineered vascular grafts must possess anti-thrombogenic property to prevent platelets activation. On the other hand, these modifications such as surface treatment with peptides must lead to a faster and efficient binding of the ECs, but not non-endothelial lineages, to the surface of scaffolds. The successful cell attachment also increases ECs growth and survival which acts as an anti-thrombogenic barrier (Fig. 4).

## Electrospun nano-fibers

**Fig. 5:**
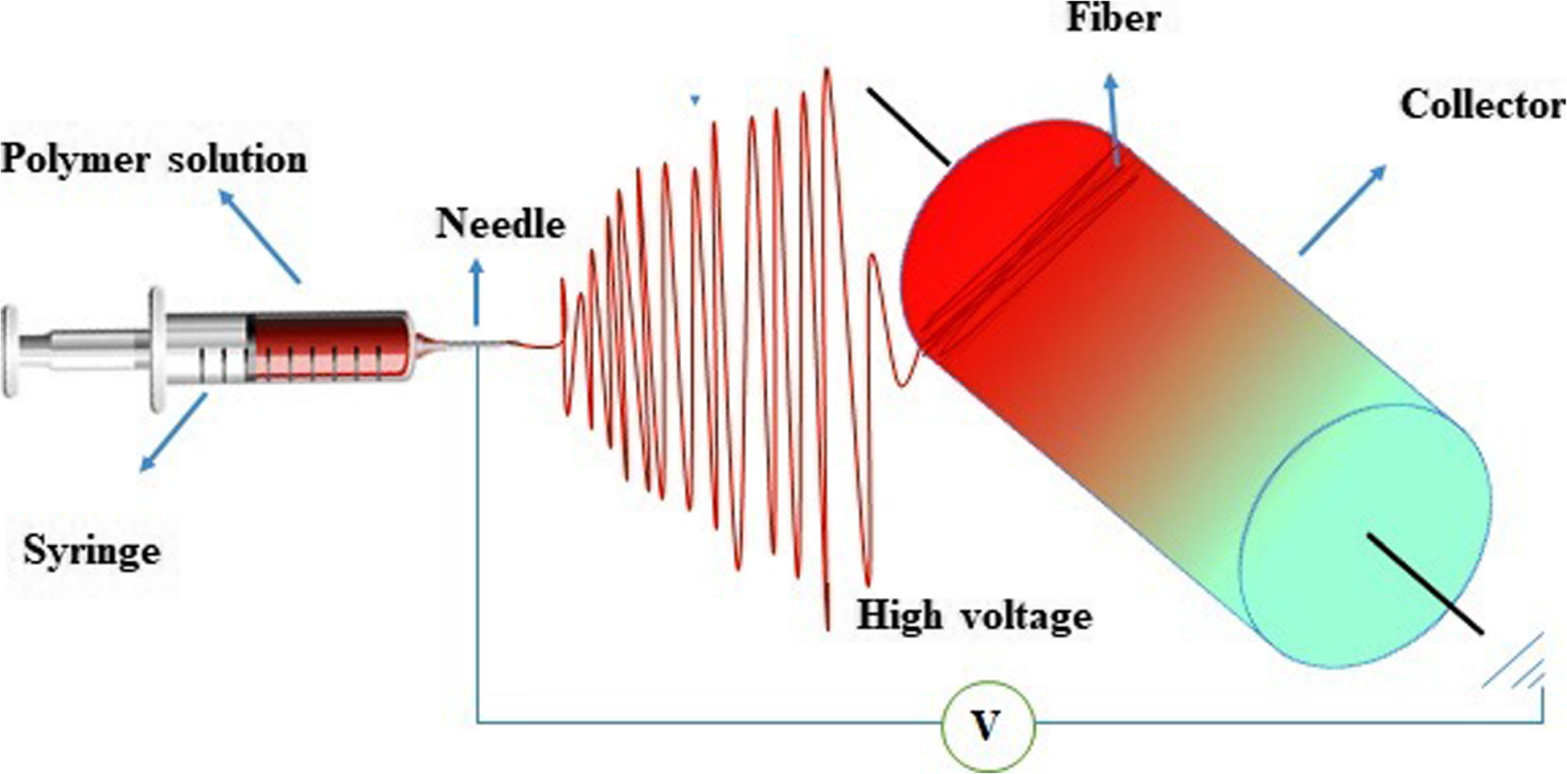
Schematic representation of the electrospinning process

In 1930, electrospinning was first introduced as a simple and economical way to fabricate very thin fibers from a polymeric solution. This technique is touted as one of the most pioneer methods of polymer nanofibers fabrication. Nanostructured scaffolds constructed by electrospinning are prone to mimic a similar condition to ECM. Electrospinning depends on the electrical forces to generate nanofibers. In this technique, nanofibers are produced from a polymeric solution or that is injected from the syringe to a region with a high electric field. When electrostatic forces overcome the surface tension of a polymeric solution, a Taylor cone is formed, and a narrow jet accelerates rapidly towards the target (collector) which is connected to the ground or with the opposite charge (Fig. 5).

### Critical factors required for designing of electrospun scaffolds

During the electrospinning process, the morphology control of synthetic polymers is easier than natural polymers. However, according to various benefits of natural polymers such as biological functions, immobilizing them onto the nanofibrous surface of synthetic polymers can be more useful. Since the phenotype and specific cellular organization are highly dependent on the entity of cell binding surface, the use of functionalized nanofibers will be beneficial [106, 108]. There are many factors and components that affect the shape and structure of electrospun nanofibers such as fiber diameter and uniformity. The structure and characteristics of the polymeric materials are affected by chemical structure, molecular weight and spatial order of the chain. In addition, the polymeric solution properties correlate with soluble concentration, viscosity, solvent type, surface tension, electrical conductivity, solvent volatility, solvent dielectric constant, and soluble temperature. To fabricate the nanofibrous scaffolds some process conditions must be considered as follows; applied voltage, spinning distance, feeding rate, the geometry of capillary tube, and collector shape. Notably, environmental factors could affect the quality of fabricated nanofibrous scaffolds. The control of humidity and environmental pressure are fundamental to scaffold synthesis [109, 110].

## The application of electrospinning for engineered vascular grafts

Based on the location and type of activity, blood vessels are different in terms of dimensions, mechanical properties, biochemical properties, cell contents, and protoplasmic elements. Therefore, all these properties should be applied in vascular tissue engineering [111]. As above-mentioned, the replacement of vessels, especially narrow vessels with a diameter of less than 6 mm, has many challenges [29]. The culture of ECs on different electrospun nanofibers has been previously studied (Table 3). Data emphasized the capability of electrospinning method to fabricate fibers capable to be used in a wide range of natural and synthetic polymers supplemented with various growth factors [67]. Except for a few cases, most studies are limited to in vivo studies. Tubular scaffolds are made using a varied range of materials. Up to now, many electrospun fibers have been manufactured using a variety of polymers, such as PLGA, elastin polyethylene oxide, PLLA, gelatin/PCL, PCL/PU, ST-gelatin, type I collagen, poly (ethylene oxide), and SPU [112].

**Table 3:** The application of electrospinning for engineered vascular grafts

Polymeric electrospun nanofibers	Cultured cell	Graft	References
Collagen	Endothelial α-SMA positive cells	Artery	[51]
Collagen type I	canine jugular α-SMA positive cells	Venous	[39, 51, 113]
PCL/collagen	Endothelial cells	Artery	[116]
PGA	Bovine aorta α-SMA positive cells	Artery	[117]
Chitosan-PCL (CS/PCL)	Human umbilical vein endothelial cell (HUVECs)	Artificial blood vessel	[120]

Studies have shown that natural polymers have potential to extremely increase cellular connectivity and penetration rate [39, 113]. In recent decades, wide experiments have been done on vascular tissue engineering by the use of electrospun scaffolds. Weinenberg and Bell were the first to develop a model of blood vessels in vitro. They fabricated a multi-layered structure from collagen that integrated with a Dacron mesh and had a similar structure to an artery with potency to endure the physiological pressure. Electron microscopic imaging revealed the existence of ECs in the luminal surface while α-SMA positive cells were located at the structure wall with the ability to acquire functional phenotype. These authors declared that ECs act as a natural barrier of permeability while produced essential factors such as vWF and prostacyclin [51].

A similar technique was used by Hirai and colleagues with similar results. They developed a tubular vascular tissue composed of collagen and vascular cells as a substitute for venous. First, type I collagen and a cold mixed solution of canine jugular α-SMA positive cells were prepared by splashing into a tubular glass mold and incubation at 37 °C. After 10 days of culturing in the medium, they achieved a dense tubular tissue. Then canine jugular ECs seeded on the lumen surface of the tissue to produce a vascular tissue with a hierarchical structure. These engineered vascular tissues that wrapped in Dacron mesh were implanted in the canine posterior vena cava for up to 24 weeks. They observed that The tissues became far dense and go forward in a time-dependent manner and is completed in about 6 months of implantation. [114, 115].

Data from an experiment conducted by Tillman et al. have been shown that electrospun PCL/collagen scaffolds could tolerate physiologic situation whenever preserve functional behavior and attachment of vessels cells. In endothelialized grafts, the reduction of platelet accumulation and hemorrhage was indicated in a rabbit model of the aortoiliac bypass. In addition, these scaffolds were able to maintain their structural integrity within a month after the implantation and these implants exhibited biomechanical endurance that was comparable to a native artery. They suggested that electrospun scaffolds composed with vascular cells may be a good alternative for vascular grafts and reconstruction. [116]. Along with the development of absorbable materials such as PGA, a lot of researches were done on vascular tissue engineering in the 1980s. The application of absorbable materials allows successful replacement with natural tissue with simultaneous degradation of biomaterials. Niklason and colleagues used biodegradable PGA tubes to cultivate bovine aorta α-SMA positive cells for 8 weeks. They also cultivated bovine ECs on the luminal surface of the scaffold. The fabricated vessel structure showed a better rupture strength compared to the native vessels. Interestingly, these structures showed an appropriate contractile response to drug stimulation [117]. In a study done by Campbell and co-workers, they examined the insertion of a silicon tube into the peritoneal cavity in rabbits and mice models. After 2 weeks, a uniform α-SMA positive cells layer was observed similar to the tunica media. Collagen surrounded the tubes as an adventitia layer with the formation of the mesothelium layer [118]. Hajiali and coworkers produced an electrospun scaffold using gelatin and PGA with different ratios (0, 10, 30 and 50 wt.%). To determine the biocompatibility of these scaffolds, they cultivated human umbilical vein endothelial cells and human umbilical artery smooth muscle cells on scaffolds and examined cell attachment and cellular viability. The results showed using PGA with 10 wt% gelatin led to enhance in the endothelial cells and the scaffold made by PGA with 30 wt% gelatin led to increase of cell adhesion, viability, and penetration in the smooth muscle cell, contrasted with the other blends. Also, by increasing the percentage of gelatin due to the intercalation between gelatin and PGA, the mechanical attributes of the scaffold were improved and the scaffolding structure was promising for use in vessel tissue engineering [119]. In another study by Fengyi et al. using chitosan and poly-caprolactone, a heparinized three-dimensional nanofibrous vascular scaffold was developed to prevent thrombosis. They used VEGF and heparin immobilization to mimic the natural blood vessel microenvironment. The results of platelet adhesion assay and activated partial thromboplastin time confirmed that Anti-thrombogenic properties of these scaffolds increased with their heparinization. Also, the adhesion and attachment of HUVECs on CS/PCL scaffold were enhanced. Therefore, the use of CS/PCL heparinized scaffolds could create a method for the production of artificial blood vessel arrays [120].

## Vasculogenic cells

Choosing the optimal cell source is one of the challenges in vascular tissue engineering. The use of autologous has always been the best choice because of the immunological responses removal. However, access to an adequate cell resource is not always available. In this regard, the use of stem cells and differentiation into distinct cell types are proposed methods. In addition to autologous stem cells, these cells could be isolated either from allogeneic or xenogeneic candidates. However, in the case of xenogeneic cells, the possibility of transmission of animal viruses is high and thereby the application of allogeneic cells is more logical.

To instruct specific cell function, manipulation is made through genetic engineering of distinct cell type or the change of ECM composition [29, 121]. It should be noted that prolonged culture period with high passage rate can affect cell phenotype. For example, isolated primary ECs can lose their phenotypes and acquire α-SMA like characteristics. To faint these effects, specific culture time and selection of appropriate culture medium are considered as more effective factors compared to cell type selection [122, 123]. Culturing cells in culture media containing VEGF and bFGF has been shown to improve differentiation and express the endothelial-derived factors such as VE-cadherin and vWF. ECs could be isolated from the human saphenous vein, umbilical vein, aorta and mediastinal fat [117, 124, 125] and bovine aorta, pulmonary arteries [126], canine saphenous vein [126]. Adult MSCs and EPCs are alternative cell sources [128, 129].

## Ideal properties of vascular grafts

The preparation of an ideal vascular graft requires such features that exist some of them are essential, others favorable. The synthetic vascular graft should have the following characteristics [130]:

- To be biocompatible
- To possess proper mechanical
- To be resistant to infection and inflammation
- To be non-thrombogenic
- To be cost-effective and be simple to use
- To an “off the shelf product” or be easily stock,
- To be accessible in different features such as diameter, length, etc.

## Conclusion

The construction of scaffolds by electrospinning for tissue engineering became a popular method every day. A great deal is the ability to build scaffolds with specific structural and functional features. Scaffolds used in vascular tissue engineering should have a layered structure in addition to biocompatibility and anticoagulant properties. Electrospun scaffolds provide a reliable matrix for cellular attachment and support their proliferation, differentiation, integrity, and phenotype. Using this technique, fibers with enhanced mechanical properties can be prepared with different combinations and different decomposition rates. However, precise control of the fiber diameter uniformity and its morphology has some defects in its functionality as an extracellular matrix. But still, the best fiber diameter and porosity between them to optimize cell function is still not fully determined. Although early studies have clearly confirmed the effectiveness of electrospun scaffolds for vascular tissue engineering, scientific literature showed a limit number of in vivo studies with the use of engineered vascular electrospun scaffolds, yet more clinical studies are needed to confirm this theory.

## Data Availability

Not applicable

## Abbreviations

2D: Two-dimensional
3D: Three-dimensional
bFGF: Basic fibroblast growth factor
ECM: Extracellular matrix
ECs: Endothelial cells
EPCs: Endothelial progenitor cells
HUVECs: Human umbilical vein endothelial cells
MSCs: Mesenchymal stem cells
PCL/Cs: Polycaprolactone/Chitosan
PGA: Polyglycolic acid
PLGA: Polylactic-co-glycolic acid
PLLA: Poly-l-lactide
SPU: Segmented polyurethane
ST-gelatin: Styrenated gelatin
VEGF: Vascular endothelial growth factor
vWF: von Willebrand’s factor
α-SMCs: Alpha-smooth muscle cells
